# Barking dogs seldom bite? a case of diagnostic overshadowing in emergency department

**DOI:** 10.1192/j.eurpsy.2021.1737

**Published:** 2021-08-13

**Authors:** J. Gonçalves Cerejeira, C. Burón, I. Santos Carrasco, C. Capella Meseguer, E. Rodríguez Vázquez, M. Queipo De Llano De La Viuda, A. Gonzaga Ramírez, G. Guerra Valera

**Affiliations:** 1 Psiquiatría, Hospital Clínico Universitario de Valladolid, Valladolid, Spain; 2 Psychiatry, Hospital Clínico Valladolid, Valladolid, Spain

**Keywords:** Stigma, Diagnostic overshadowing, factitious disorder

## Abstract

**Introduction:**

Diagnostic overshadowing is one of the main consequences of stigma involving patients diagnosed with a psychiatric disorder. Some studies show that in emergency departments, being diagnosed with a psychiatric illness can lead to a poor evaluation of organic symptoms, delaying the diagnosis and putting the patient’s life at risk.

**Objectives:**

- To present the case of a patient diagnosed with factitious disorder who was misdiagnosed after attending the emergency department due to the stigma related to his psychiatric diagnosis. - To provide a reflection on stigma in mental health.

**Methods:**

We will present a case report and a literature review.

**Results:**

We report a case of a 57-year-old man diagnosed with a factitious disorder. He attended the emergency department of our tertiary care center with confused speech, desorientation and disruptive behavior at home. Although the clinical picture was compatible with a confusional state, he was ordered to be admitted to the psychiatric service. No blood test was previously requested. Three hours after being admitted, he suffered an episode of seizures. A blood test was requested and severe hypomagnesemia (0.2 mg / dl) was found. Because of this episode the patient was admitted to the Intensive Care Unit for three days.

**Conclusions:**

Factitious disorder is a serious mental disorder with a significant stigmatizing burden. Giving a patient this diagnostic label should be the subject of careful thought in order to protect him from future diagnostic neglect.

**Disclosure:**

No significant relationships.

